# Supplementary material for: Understanding the influence of ethnicity on adherence to antidiabetic medication: Meta-ethnography and systematic review
Source: PLoS One. 2023 Oct 12;18(10):e0292581. doi: 10.1371/journal.pone.0292581 (PMC10569585; doi:10.1371/journal.pone.0292581)
Supplement: S1 File — (ZIP) [file pone.0292581.s001.zip › S3 Table. .docx]

**S3 Table. Results of the risk of bias assessment of the included studies**

| Critical Appraisal Skills Tool Screening Questions | **Study citation** | | | | | | | | | | | | | | | | | | | | |
| --- | --- | --- | --- | --- | --- | --- | --- | --- | --- | --- | --- | --- | --- | --- | --- | --- | --- | --- | --- | --- | --- |
|  | (Jamil et al.,2022) | (Timsina et al., 2022) | (Parkin et al., 2021) | (Ahmad et al.,2021) | (Omodara et al.,2021) | (Pardhan et al.,2020) | (de-Graft Aikins et al., 2019) | (Jaam et al.,2018) | (Shiyanbola et al., 2018) | (Sapkota et al.,2018) | (Bockwoldt et al.,2017) | (Patel et al.,2016) | (Joo & Lee, 2016) | (Peeters et al., 2015) | (Mohan et al.,2013) | (Lynch et al., 2012) | (Singh et al., 2012) | (Barko et al.,2011) | (Noakes,2010) | (Ho & James ,2006) | (Lawton et al.,2005) |
| 1- Was there a clear statement of the aims of the research? | Yes | Yes | Yes | Yes | Yes | Yes | Yes | Yes | Yes | Yes | Yes | Yes | Yes | Yes | Yes | Yes | Yes | Yes | Yes | Yes | Yes |
| 2- Is qualitative methodology appropriate? | Yes | Yes | Yes | Yes | Yes | Yes | Yes | Yes | Yes | Yes | Yes | Yes | Yes | Yes | Yes | Yes | Yes | Yes | Yes | Yes | Yes |
| 3- Was the research design appropriate to address the aims of the research? | Yes | Yes | Yes | Yes | Yes | Yes | Yes | Yes | Yes | No | No | No | Yes | No | No | Yes | No | No | No | No | Yes |
| 4- Was the recruitment strategy appropriate to the aims of the research? | Yes | Yes | Yes | Yes | Yes | Yes | Yes | Yes | Yes | No | Yes | Yes | Yes | Yes | Yes | Yes | Yes | Yes | Yes | Yes | Yes |
| 5-Was the data collected in a way that addressed the research issue? | Yes | Yes | Can’t tell | Yes | Yes | Yes | Yes | Yes | Yes | Yes | Yes | Yes | Yes | Yes | Yes | Yes | Yes | Yes | Yes | Yes | Yes |
| 6- Has the relationship between researcher and participants been adequately considered? | Can’t tell | Can’t tell | Can’t tell | Can’t tell | Can’t tell | Can’t tell | Can’t tell | Can’t tell | Can’t tell | Can’t tell | Can’t tell | Can’t tell | Can’t tell | Can’t tell | Can’t tell | Can’t tell | Can’t tell | Yes | Can’t tell | Yes | Can’t tell |
| 7- Have ethical issues been taken into consideration? | Yes | Yes | Yes | Yes | Can’t tell | Yes | Yes | Yes | Yes | Yes | Yes | Yes | Yes | Yes | Yes | Yes | Yes | Yes | Yes | Yes | No |
| 8- Was the data analysis sufficiently rigorous? | Yes | Yes | Yes | Yes | Yes | Yes | Yes | Yes | Yes | No | Yes | Yes | Yes | Yes | Yes | Yes | Yes | Yes | Yes | Yes | Yes |
| 9- Is there a clear statement of findings? | Yes | Yes | Yes | Yes | Yes | Yes | Yes | Yes | Yes | Yes | Yes | Yes | Yes | Yes | Yes | Yes | Yes | Yes | Yes | No | Yes |
| 10- How valuable is the research? | Yes | Yes | Yes | Yes | Yes | Yes | Yes | Yes | Yes | Yes | Yes | Yes | Yes | Yes | Yes | Yes | Yes | Yes | Yes | Yes | Yes |
| Comments | - | - | Q5: No discussion around data saturation | - | Q7: No statement around approval of the ethics committee | Q5: No discussion around data saturation | - | - | - | - | - | - | - | - | - | - | - | - | - | - | - |
