# Supplementary material for: Understanding the influence of ethnicity on adherence to antidiabetic medication: Meta-ethnography and systematic review
Source: PLoS One. 2023 Oct 12;18(10):e0292581. doi: 10.1371/journal.pone.0292581 (PMC10569585; doi:10.1371/journal.pone.0292581)
Supplement: S1 File — (ZIP) [file pone.0292581.s001.zip › S4 Table, S5 Table, and S6 Table..docx]

**S4 Table, S5 Table, and S6 Table. Result of the qualitative data synthesis**

**S4 Table: Theme 1: Cultural underpinnings**

| **Synthesised themes (third order constructs)** | **Sub-themes** | **Second order constructs: the authors interpretations of the original findings** | **First Order constructs: examples of direct quotations from the participants of the study** |
| --- | --- | --- | --- |
| **CULTURAL UNDERPINNINGS** | ***Preference for alternative medicines*** | Efficacy of alternative remedies | *‘If I keep using the herbs, I’m using now, which I know it’s working for me, I know I will soon stop taking my medication completely’. (Omodara et al., 2021)*  *Black sub-Saharan African* |
|  |  |  | *‘I believe in traditional remedies, and I know some of them works for diabetes. My mother has diabetes as well, and she usually uses herbs and bitter kola …… She usually gets from Nigeria to manage her sugar level, so I use it as well’. (Omodara et al., 2021)*  *Black sub-Saharan African* |
|  |  |  | *‘I use Aloe Vera to lower my blood sugar…...’. (Omodara et al., 2021)*  *Black sub-Saharan African* |
|  |  | Alternative treatments | *‘When I first became a diabetic, I didn’t consult with a doctor, I took a treatment of nopal that a nutritionist gave me, and this immediately made me lose weight’. (Lynch et al., 2012)*  *Mexican American* |
|  |  | Religious beliefs and spirituality | *‘It is part of our tradition in South Africa; we use oils, plants, and herbs for treatment alongside prayers……’. (Omodara et al., 2021)*  *Black sub-Saharan African* |
|  |  | Initiation of antidiabetic medications | *‘I avoided (insulin) for almost 2–3 years…That time once I had taken Ramdev’s (Ayurvedic) medication…’. (Sapkota et al., 2018) Nepalese* |
|  |  |  | *‘…. Let’s say because of the family pressure I started from there (Ayurvedic medications)’. (Sapkota et al., 2018)*  *Nepalese* |
|  |  |  | *‘My relatives from Nepal suggested me to take Ram Dev’s (Ayurvedic) medications … then after I started taking them’. (Sapkota et al., 2018)*  *Nepalese* |
|  |  | Implementation of antidiabetic medications | *‘if you take this English medicine, there will be side effect …. You should not take English medicines…’. (Sapkota et al., 2018)*  *Nepalese* |
|  |  | Long term cessation of antidiabetic medications | *‘I took the medication for 1 month,…..Yes, then I started on herbs. I kept myself on herbs for 4 years’. (Sapkota et al., 2018)*  *Nepalese* |
|  |  | Adherence to medication | *‘I take bitter gourd with pieces in water and aloe veera’. (Pardhan et al., 2020)*  *South Asian* |
|  |  | Social network and social support | *‘Lots of people in the family have it so they used to tell me…. try karela juice…’. (Patel et al., 2016)*  *British South Asian* |
|  |  | Beliefs about oral hypoglycaemic agents | *‘They (family members) told me that karabaş would be good. My son has brought these herbs from Turkey. I boiled and drank them but it didn’t help…’.(Peeters et al., 2015)*  *Turkish* |
|  |  | International travel to visit friends and relatives | *‘I take [name of country] medicine. When I am in [name of the country], I take it because it is more effective and has fewer side effects’. (Jaam et al., 2018)*  Sri Lankan |
|  |  |  | *‘Our grandparents, they teach us these are good and they come from nature, so they are good’. (Jaam et al., 2018)*  Sri Lankan |
|  |  | Fear of medication side-effects | ‘*….it was suggested by my brother to take ayurvedic medication, he told that its really effective and no side effects’. (Ahmad et al., 2021)*  *Indian* |
|  |  |  | *‘... my friend brought from India ayurvedic medication.... I didn’t see much benefit out of it’. (Ahmad et al., 2021)*  *Indian* |
|  |  |  | ‘*... 1 year no medication taking and manage well with his healthy diet, exercise and meditation with some herbs’. (Ahmad et al., 2021)*  *Indian* |
|  |  | Fear of side effects and medication dependency | ‘*Want to be away from all these strong drugs [because of side effects] and make addicted.... going to start natural method such as ayurvedic medication’.(Ahmad et al., 2021)*  *Indian* |
|  |  | Negative belief about medicines | ‘*My colleague in Australia said allopathic medications have lots of side effects…. she insisted to take ayurvedic medication because her mother using it and benefitted in India, ... , so I decide to initiate ayurvedic medication instead of metformin’. (Ahmad et al., 2021)*  *Indian* |
|  |  | Distrust of Western medicine | *‘…. Chinese herbalist would weigh each serving. So, Western medicine is powerful, best to be avoided’.(Ho & James, 2006)*  *Chinese* |
|  |  |  | *‘They always say Western medicine makes you very weak’. (Ho & James, 2006)*  *Chinese* |
|  |  |  | *‘How long has insulin been around?.... versus 2000 years of Chinese history…’.(Ho & James, 2006)*  *Chinese* |
|  |  |  | *‘….When I talk to my herbal doctor, he says Chinese medicine restores the balance of your system’.(Ho & James, 2006)*  *Chinese* |
|  |  | Self-management of diabetes | *‘I want to know about herbs because my doctor gives me pills, but they are all chemical based. They cause me to either sleep too much, or not enough. My stomach and liver started to hurt’.(Barko et al., 2011)*  Russian-speaking Slavic immigrant American |
|  |  | Complementary and alternative medicines augmented Western medicines | *‘….people say, cinnamon isvery good for diabetes and tamarind is very good, so one time I start taking those.. ’.* (*Jamil et al., 2022)*  *South Asian* |
|  |  | Medication management | *‘Herbal medicine is very effective in treating diabetes, when you take the western medicine in excess you can get other complications’.* (*de-Graft Aikins et al.,2019)*  *Ghanaian migrants* |
|  | ***Perspective of prescribed medicine*** | Perceptions of oral hypoglycaemic agents (OHA) | *‘See, in Pakistan, the medications are not right, they’re just a waste of time, waste of money. I mean these [referring to OHAs] are the real stuff. These are what really work’.(Lawton et al., 2005)*  *British Pakistani* |
|  |  |  | *‘I don’t think you can get the same kinds of medicine that you can get here, you know, like metformin. This is one of the most important drugs to take for it’. (Lawton et al., 2005)*  *British Indian* |
|  | ***Social stigma*** | Social stigma and other cultural pressures | *‘The biggest obstacle is the stigma around diabetes. There are not a lot of people, family friends who know that we (brothers) are diabetic……. no one is going to marry him because he is a diabetic’. (Singh et al., 2012)*  *South Asian* |
|  |  |  | ‘*… I mean you try and hide your illness; no one should know that you have any illness’. (Singh et al., 2012)*  *South Asian* |
|  |  |  | *‘… it is more because of our culture and community. People look at you and go, ‘Oh God! Is he taking insulin?’ … people feel that you have a very dangerous kind of disease …it is really embarrassing’. (Singh et al., 2012)*  *South Asian* |
|  |  |  | *‘If I am going around somebody’s house for a meal, they make me do the injection before I go’. (Singh et al., 2012)*  *South Asian* |
|  |  | Culture | *‘The reason why my mother suffers with it, is that she is a very conservative, proud woman. She finds it hard to tell people she’s on insulin’. (Noakes, 2010)*  Black African |
|  |  | Social pressure | *‘she got married and pregnant, and she did not tell her husband she is diabetic’. (Jaam et al., 2018)* |
|  |  |  | ‘*Honestly, I intentionally sometimes not take [injections] it in front of people […] I remember I was once taking insulin, and someone got up […] and from far he screamed […] hey heroine!’. (Jaam et al., 2018)* |
|  |  | Stigma related diabetes | ‘*Initially I met my GP in Australia and [was] diagnosed with type 2 diabetes and started with metformin but I did not inform my wife, and even to my parents that I am diabetic, So I do not take medicine in front of her [wife]...... wherever we go in social or religious gatherings, I used to take same food as no one knew that I am diabetic ... the problem in Indian community they will show pity’. (Ahmad et al., 2021)*  *Indian migrant* |
|  |  | Greater familiarity with/easier access to traditional Chinese medicine | ‘*The more Chinese you are, you’re really more attuned to what you can take and how to go about taking it, and how regular and so forth’. (Ho & James, 2006)*  *Chinese* |
|  |  |  | ‘ *[Chinese patients] go for certain herbal medicines just to get away from the need to receive needles’. (Ho & James, 2006)*  *Chinese* |
|  |  | Cultural factors contributing to barriers | ‘ *My family views people taking insulin as more of a handicap….’. (Ho & James, 2006)*  *Chinese* |
|  |  | Social stigma | ‘Once people see you are taking insulin, especially in my culture and community [sigh] they put on this sympathy look; people think you have a killable disease’. (Omodara et al., 2021)  *Black sub-Saharan African* |
|  |  |  | *‘Diabetes is a big stigma-related problem in our community, especially among the Black African community…’.*  *(Omodara et al., 2021)*  *Black sub-Saharan African* |
|  | ***Family and social support*** | Perceived solutions for improving adherence | *‘[…] my church try to bring somebody in to talk to us about it (diabetes) […] ……come and meet and just sit down and discuss the problems that you’re having with your diabetes. Maybe somebody can help you’. (Shiyanbola et al., 2018)*  *African American* |
|  |  |  | *‘[…] in the high black areas, in the neighbourhood centers, they bring in people […] they try to help them to*  *be a self-advocate for themselves. They teach them that if you’re going to stay healthy, you’re going to have to do*  *it […] you’ve got to put the work into it to find out, what is happening with your body and what it is that the black people experience versus the white people’s experience and the doctors’. (Shiyanbola et al., 2018)*  *African American* |
|  |  |  | *‘…Knowledge is power, and you have to have a support group…’. (Shiyanbola et al., 2018)*  *African American* |
|  |  | Difficulty in knowing how to access and understand the available information | *‘I don’t know who to ask – I sometimes ask my friend who is also a diabetic’.*  *(Pardhan et al., 2020)*  *South Asian* |
|  |  | Limited role perceived for GPs/Practice | ‘My family and friends can support me when there are new developments, like my cousin called me last week to inform me about new insulin which you only have to take once and told me to ask my GP...the GP doesn’t have enough time…’. (Patel et al., 2016)  *British Pakistani* |
|  |  | Social networks and social support | *‘….when I go to the mosque to pray other people that have diabetes they talk about it…’. (Patel et al., 2016)*  *British Bangladeshi* |
|  |  | Immediate/close family members | *‘I feel there is no life without wife. After a certain age there is a desperate need for a partner … they will remind you and say, ‘have you taken your insulin?’. (Singh et al., 2012)*  *South Asian* |
|  |  | The message | *‘They’ll welcome you more because they will see you as one of theirs and you’re not likely to lie to them, you know … And because of that culture thing whereby they don’t open up enough to see their own kind being open they will feel relaxed’.(Noakes, 2010)*  *Black African* |
|  |  | Social support | *‘.. (…) When I travel to Turkey my daughter is always with me. She takes care of my insulin and my pills’. (Peeters et al., 2015)*  *Turkish* |
|  |  | Open communication improved medication adherence | *‘…..Daughters remind me‘did you take it?..... The other day, I ate out with friends and then forgot to take my medicine’*  (*Jamil et al., 2022)*  *South Asian* |
|  | ***Religious beliefs and practices*** | Patients’ individual characteristics | *‘There is one-month fasting but not complete; we can use vegetable, not non-vegetable. Light food and I do not use insulin. Only vegetable, so I do not think this will*  *increase my sugar’. (Jaam et al., 2018)*  *Indian* |
|  |  |  | *‘I change the dose in Ramadan by myself […] It’s two times […] morning and evening same […]’. (Jaam et al., 2018)*  *Pakastani* |
|  |  | Religiosity in combination with psychological problems | *‘There is nothing you can do about it. I’m religious, everything comes from Allah. So there is nothing you can do about it’. (Peeters et al., 2015)*  *Turkish* |
|  |  | Beliefs about diabetes | *‘Of course, I accept it (his diabetes). Because Allah gave it to me. My faith makes it easier to deal with it’. (Peeters et al., 2015)*  *Turkish* |
|  |  | Adjustment of oral hypoglycaemic agents during Ramadan fasting | *‘ I: ‘But you have to take three pills (OHA) a day?’ ‘I take one at sun dawn and one at sunset.’*  *I: ‘Does your doctor know? ’I decide this myself’. (Peeters et al., 2015)*  *Turkish* |
|  |  | Supportive relationships have an impact on medication adherence | *‘I’m gonna say that my faith helps me get through the hardship of being a diabetic’. (Bockwoldt et al., 2017)*  *African American* |
|  |  | Religion | *‘…. Whatever the doctor or nurses prescribed is helped through God’s mercy. Sometimes it not helps everybody, but God isn’t in control of these things … As far as he concerned, God helps me through them’. (Noakes, 2010)*  *African-Caribbean* |
|  |  |  | *‘I know there is people that would say they can’t do insulin because they say they would rather God help them’. (Noakes, 2010)*  *African-Caribbean* |
|  |  | Religious beliefs and values | *‘There is a lot of power in a prayer. He has given me this condition so he knows about it’. (Singh et al., 2012)*  *South Asian* |
|  |  |  | *‘Prayers give me a lot of support……Prayers have a lot of healing power’. (Singh et al., 2012)*  *South Asian* |
|  |  | Belief in health care | *‘God gave you six senses. Right? And so if the doctor tell you you sick, and he tell you you must take this medicine to stay alive, better use your common sense. It’s a wonderful thing to pray and have faith, but God gave you six senses. He told you to listen and learn’. (Shiyanbola et al., 2018)*  *African American* |
|  |  | Religious beliefs and spirituality | *‘You put everything in prayers. Even though I don’t use medication to control my glucose level, I still put other remedies I use in prayer’. (Omodara et al., 2021)*  *Black sub-Saharan African* |
|  |  |  | *‘Most times when I fast and pray, I always feel better with my diabetes’.*  *(Omodara et al., 2021)*  *Black sub-Saharan African* |
|  |  |  | *‘Prayer is my main key to my strength and managing my diabetes…’.*  *(Omodara et al., 2021)*  *Black sub-Saharan African* |
|  |  | Faith-based coping | *‘I know prayer can cure diabetes; it all depends on the faith you have’.* (*de-Graft Aikins et al.,2019)*  *Ghanaian migrants* |

**S5 Table: Theme 2: Communicating and building relationship**

| **Synthesised themes (third order constructs)** | **Sub-themes** | **Second order constructs: the authors interpretations of the original findings** | **First Order constructs: examples of direct quotations from the participants of the study** |
| --- | --- | --- | --- |
| **COMMUNITING AND BUILDING RELATIONSHIP** | *Language and communication* | Patient–provider communication barriers | *‘There are those who have language issues, so they do not understand, they receive the medicine, but they do not know how to take it’. (Jaam et al., 2018)* |
|  |  | Language issues | ‘…..But it is still difficult to talk in English about my diabetes symptoms and questions’. (Joo & Lee, 2016)  *Korean American immigrant* |
|  |  | Limited role perceived for GPs/Practice | *‘My GP in Pakistan I talk to him and get advice*  *over the phone about my diabetes’. (Patel et al., 2016)*  *British Pakistani* |
|  |  | Challenges to proper medication taking | *‘The problem is that, for example....for someone who does not speak adequate English who goes to a doctor, how can you explain yourself to the physician?’. (Mohan et al., 2013)*  *Latino* |
|  |  | Language barriers | ‘I sometimes can’t understand what the GP/nurse is explaining about my diabetes as they do not speak Urdu’. (Pardhan et al., 2020)  *South Asian* |
|  |  | Lack of information from health care providers | *‘I sometimes can’t understand what the GP is explaining’. (Pardhan et al., 2020)*  *South Asian* |
|  |  | Access to diabetes-related information | *‘We find it difficult to understand English. Information (is) not provided in local language’. (Pardhan et al., 2020)*  *South Asian* |
|  |  | Healthcare team and diabetes-related literature | *‘… the SA nurse can understand our language and she is a very nice lady. She is worth all the praise. Doctors here talk in English … he (a consultant) is also nice but most of the time he cannot understand my English’. (Singh et al., 2012)*  *South Asian* |
|  |  | Patient–health care provider relationship | *‘‘I did (want to ask for more information) but I don’t know the language.’’(Peeters et al., 2015)*  *Turkish* |
|  |  | Medicines | *‘I have tried to get it on the phone, but they never answer... a machine answers in English and I don’t understand anything’. (Lynch et al., 2012)*  *Mexican American* |
|  |  | Family and community support invaluable for health | *‘Our doctor helps a lot. We are so happy that he can speak and understand our language (Nepalese language) ’.*  *(Timsina et al.,2022)*  *Bhutanese* |
|  | *Spending time* | Seeing multiple physicians/care provider | *‘I saw so many doctors here. I once told my friend […] I told him in [name of the clinic], it’s like the United Nations […]’. (Jaam et al., 2018)* |
|  |  | Patient–provider communication barriers | *‘There is not really enough time, actually there is never enough time, but you also can’t do it all in one visit’. (Jaam et al., 2018)* |
|  |  |  | *‘There is no time to even deal with him [the patient] much, given the pressure here in this center’. (Jaam et al., 2018)* |
|  |  | Perceived solutions for improving adherence | *‘Get educated about black people. Education about the clientele you serve. So you have to know the cultures that you serve […]’. (Shiyanbola et al., 2018)*  *African American* |
|  |  | Limited role perceived for GPs/Practice. | *‘...you can’t blame them because they are seeing so many patients a day, they haven’t got the time to spend 20 minutes or half an hour to talk and tell you things....’. (Patel et al., 2016)*  *South Asian* |
|  |  | Language barriers | *‘….they won’t tell you anything in depth as appointments are limited to only few minutes’. (Pardhan et al., 2020)*  *South Asian* |

**S6 Table: Theme 4: Managing diabetes at home and away**

| **Synthesised themes (third order constructs)** | **Sub-themes** | **Second order constructs: the authors interpretations of the original findings** | **First Order constructs: examples of direct quotations from the participants of the study** |
| --- | --- | --- | --- |
| MANGING DIABETES AT HOME AND AWAY | *Practicalities* | Beliefs about diet and diabetes management | *‘….no electricity for about 8 hours...so in the summer it’s very difficult and because I take insulin I have nowhere…’. (Patel et al., 2016)*  *British Pakistani* |
|  |  | Healthcare system | *‘…. And it’s expensive and you have to keep it in the fridge. In Jamaica some people don’t even have a fridge in the house’. (Noakes, 2010)*  *African-Caribbean* |
|  | *Views and perspective* | Changing time zones | *‘…some days I was completely forgetting them for days on end. Cause you were travelling for 24 hours’. (Parkin et al., 2021)*  *Non-Māori non-Pacific* |
|  |  | Beliefs about diet and diabetes management | *‘When I go there my diabetes is gone....’. (Patel et al., 2016)*  *British Pakistani* |
|  |  |  | *‘ I: Did you take all your medication with you?*  *...I never take any medicine when I am there...for 6 or 7 weeks that I am there, I never take’. (Patel et al., 2016)*  *British Pakistani* |
|  |  | Impact of causal beliefs about diabetes (stress, Belgian climate) on medication adherence | *‘In Turkey I don’t take it (his OHA) at all. I sometimes measure my sugar and it is always lower than (when I am) over here’. (Peeters et al., 2015)*  *Turkish* |
|  |  |  | *‘Once a year I go to Turkey for 5 to 6 weeks. Sometimes I don’t inject (the insulin) at noon although I eat and drink a lot. Because I have no worries my sugar (blood sugar level) is very low’. (Peeters et al., 2015)*  *Turkish* |
|  |  | International travel to visit friends and relatives | *‘He [patient] has an idea that when traveling to his home country, he will get better and his health will improve’. (Jaam et al., 2018)* |
