# Supplementary material for: Understanding the influence of ethnicity on adherence to antidiabetic medication: Meta-ethnography and systematic review
Source: PLoS One. 2023 Oct 12;18(10):e0292581. doi: 10.1371/journal.pone.0292581 (PMC10569585; doi:10.1371/journal.pone.0292581)
Supplement: S1 File — (ZIP) [file pone.0292581.s001.zip › S7 Table..docx]

**S7 Table. Data extraction form.**

| Study citation | Study aim | Setting | Participants | | | | | | Recruitment/data collection& analysis |
| --- | --- | --- | --- | --- | --- | --- | --- | --- | --- |
|  |  |  | Sample size | Gender/sex | Age: Mean ± SD/median±Range | Ethnic minority group | Type of diabetes | Antidiabetic medication |  |
|  |  |  |  |  |  |  |  |  |  |
