# Supplementary material for: Understanding the influence of ethnicity on adherence to antidiabetic medication: Meta-ethnography and systematic review
Source: PLoS One. 2023 Oct 12;18(10):e0292581. doi: 10.1371/journal.pone.0292581 (PMC10569585; doi:10.1371/journal.pone.0292581)
Supplement: S1 File — (ZIP) [file pone.0292581.s001.zip › S8 Table.docx]

| **Review Finding**  **S8 Table. CERQual assessments** | | **CERQual**  **Assessment of**  **Confidence in the**  **Evidence** | **Explanation of CERQual**  **Assessment** | **Studies**  **Contributing to**  **the Review**  **Finding** |
| --- | --- | --- | --- | --- |
| ***Finding 1:***  Cultural underpinnings | Perspectives of prescribed medicine and preferences for alternatives | High confidence. | **Assessment of methodological limitation**  Minor concerns because most of the studies were unclear regarding relationship between researchers and participants, one study had unclear recruitment strategy, and one paper didn’t provide in-depth description of the analysis process. | [1-12] |
|  |  |  | **Assessment of coherence**  No/very minor concerns, as the studies had clear underlying data supporting the finding. |  |
|  |  |  | **Assessment of adequacy**  Minor concerns regarding adequacy because there were 12 studies out of 21 supporting the finding. |  |
|  |  |  | **Assessment of relevance**  Very minor concerns, as one study included participants from low-income countries and high-income countries. |  |
|  | Social stigma of the condition | Moderate confidence. | **Assessment of methodological limitation**  Minor concerns because most of the studies were unclear regarding relationship between researchers and participants, and one study had no clear  statement of findings. | [7, 8, 10, 13, 14] |
|  |  |  | **Assessment of coherence**  No/very minor concerns as there were clear data supporting the finding. |  |
|  |  |  | **Assessment of adequacy**  Moderate concerns because only five studies contributing to this finding. |  |
|  |  |  | **Assessment of relevance**  Very minor concerns, as this finding covered different settings and different ethnic groups. |  |
|  | Family and social support | High confidence. | **Assessment of methodological limitation**  Minor concerns because most of the studies were unclear regarding relationship between researchers and participants. | [5, 8, 11, 13, 15, 16] |
|  |  |  | **Assessment of coherence**  No/very minor concerns, as the studies had clear underlying data supporting the finding. |  |
|  |  |  | **Assessment of adequacy**  Minor concerns because six studies contributing to this finding. |  |
|  |  |  | **Assessment of relevance**  Minor concerns, as four out of six studies contributing to this finding conducted in one high income country. |  |
|  | Religious beliefs and practices | High confidence. | **Assessment of methodological limitation**  Minor concerns because most of the studies were unclear regarding relationship between researchers and participants, and one study was unclear regarding ethical considerations. | [3, 4, 7, 8, 12, 13, 15, 17] |
|  |  |  | **Assessment of coherence**  No/very minor concerns, as the studies had clear underlying data supporting the finding. |  |
|  |  |  | **Assessment of adequacy**  Minor concerns regarding adequacy because there were 8 studies out of 21 supporting the finding. |  |
|  |  |  | **Assessment of relevance**  Very minor concerns, as this finding covered different settings and different ethnic groups. |  |
| ***Finding 2:***  *Communication* | Language barrier | Moderate confidence. | **Assessment of methodological limitation**  Minor concerns because most of the studies were unclear regarding relationship between researchers and participants. | [7, 11] |
|  |  |  | **Assessment of coherence**  No/very minor concerns, as the studies had clear underlying data supporting the finding. |  |
|  |  |  | **Assessment of adequacy**  Moderate concerns because only two studies contributing to this finding. |  |
|  |  |  | **Assessment of relevance**  Very minor concerns, as this finding covered different settings and different ethnic groups. |  |
|  | Building relationships | Moderate confidence. | **Assessment of methodological limitation**  Minor concerns because most of the studies were unclear regarding relationship between researchers and participants. | [5, 7, 11] |
|  |  |  | **Assessment of coherence**  No/very minor concerns, as the studies had clear underlying data supporting the finding. |  |
|  |  |  | **Assessment of adequacy**  Moderate concerns because only three studies contributing to this finding. |  |
|  |  |  | **Assessment of relevance**  Minor concerns, as two out of three studies contributing to this finding conducted in one high income country. |  |
| ***Finding 3:***  *Managing diabetes during visiting home countries* | | High confidence. | **Assessment of methodological limitation**  Minor concerns because most of the studies were unclear regarding relationship between researchers and participants, and one study had unclear statement of data collection. | [3, 5, 7, 9, 13] |
|  |  |  | **Assessment of coherence**  No/very minor concerns, as the studies had clear underlying data supporting the finding. |  |
|  |  |  | **Assessment of adequacy**  Minor concerns because five studies contributing to this finding. |  |
|  |  |  | **Assessment of relevance**  Very minor concerns, as this finding covered different settings and different ethnic groups. |  |

**References**

1. Timsina MK, Peltzer JN, Pokharel Y, Peterson JM, Schwartz LJ, LeMaster JW. Understanding Medication Adherence in Bhutanese Refugees With Diabetes in a Midwestern City. Journal of Transcultural Nursing. 2022;33(3):324-33.

2. Sapkota S, Brien J-aE, Aslani P. Nepalese patients’ anti-diabetic medication taking behaviour: an exploratory study. Ethnicity & health. 2018;23(7):718-36.

3. Peeters B, Van Tongelen I, Duran Z, Yüksel G, Mehuys E, Willems S, et al. Understanding medication adherence among patients of Turkish descent with type 2 diabetes: a qualitative study. Ethnicity & Health. 2015;20(1):87-105.

4. de-Graft Aikins A, Dodoo F, Awuah RB, Owusu-Dabo E, Addo J, Nicolaou M, et al. Knowledge and perceptions of type 2 diabetes among Ghanaian migrants in three European countries and Ghanaians in rural and urban Ghana: The RODAM qualitative study. PloS one. 2019;14(4):e0214501.

5. Patel NR, Kennedy A, Blickem C, Reeves D, Chew-Graham C. “I’m Managing My Diabetes between Two Worlds”: Beliefs and Experiences of Diabetes Management in British South Asians on Holiday in the East—A Qualitative Study. Journal of Diabetes Research. 2016;2016.

6. Barko R, Corbett CF, Allen CB, Shultz JA. Perceptions of diabetes symptoms and self-management strategies: a cross-cultural comparison. Journal of transcultural nursing. 2011;22(3):274-81.

7. Jaam M, Hadi MA, Kheir N, Ibrahim MIM, Diab MI, Al-Abdulla SA, et al. A qualitative exploration of barriers to medication adherence among patients with uncontrolled diabetes in Qatar: integrating perspectives of patients and health care providers. Patient preference and adherence. 2018;12:2205.

8. Singh H, Cinnirella M, Bradley C. Support systems for and barriers to diabetes management in South Asians and Whites in the UK: qualitative study of patients' perspectives. BMJ Open. 2012;2(6):e001459.

9. Parkin L, Maclennan K, Te Morenga L, Inder M, Moata'ane L. What helps and hinders metformin adherence and persistence? A qualitative study exploring the views of people with type 2 diabetes. The New Zealand Medical Journal (Online). 2021;134(1536):25-40.

10. Ahmad A, Khan MU, Aslani P. A Qualitative Study on Medication Taking Behaviour Among People With Diabetes in Australia. Frontiers in pharmacology. 2021;12.

11. Pardhan S, Nakafero G, Raman R, Sapkota R. Barriers to diabetes awareness and self-help are influenced by people's demographics: perspectives of South Asians with type 2 diabetes. Ethnicity & health. 2020;25(6):843-61.

12. Bockwoldt D, Staffileno BA, Coke L, Hamilton R, Fogg L, Calvin D, et al. Understanding experiences of diabetes medications among African Americans living with type 2 diabetes. Journal of Transcultural Nursing. 2017;28(4):363-71.

13. Noakes H. Perceptions of black African and African-Caribbean people regarding insulin. J Diabetes Nurs. 2010;14(4):148-56.

14. Ho EY, James J. Cultural barriers to initiating insulin therapy in Chinese people with type 2 diabetes living in Canada. Canadian Journal of Diabetes. 2006;30(4):390-6.

15. Shiyanbola OO, Brown CM, Ward EC. “I did not want to take that medicine”: African-Americans’ reasons for diabetes medication nonadherence and perceived solutions for enhancing adherence. Patient preference and adherence. 2018;12:409.

16. Peeters B, Van Tongelen I, Duran Z, Yuksel G, Mehuys E, Willems S, et al. Understanding medication adherence among patients of Turkish descent with type 2 diabetes: a qualitative study. Ethnicity & Health.20(1):87-105.

17. Omodara D, Gibson L, Bowpitt G. Exploring the impact of cultural beliefs in the self-management of type 2 diabetes among Black sub-Saharan Africans in the UK–a qualitative study informed by the PEN-3 cultural model. Ethnicity & health. 2021:1-19.
