# Supplementary material for: Understanding the influence of ethnicity on adherence to antidiabetic medication: Meta-ethnography and systematic review
Source: PLoS One. 2023 Oct 12;18(10):e0292581. doi: 10.1371/journal.pone.0292581 (PMC10569585; doi:10.1371/journal.pone.0292581)
Supplement: S1 Text — (DOCX) [file pone.0292581.s002.docx]

**S1 Text: Database search terms**

1. **Medline, Embase, CINAHL, and PsycINFO databases**

Full search = (Diabetes) AND (Medication adherence) AND/OR (Glycaemic control) AND/OR (Ethnicity) AND (Qualitative methodology) AND/OR (Barriers or facilitators)

Diabetes:

(diabetes.mp. or diabetes mellitus/OR type 1 diabetes.mp. or insulin dependent diabetes mellitus OR type 2 diabetes.mp. or non-insulin dependent diabetes mellitus/OR exp Diabetes Mellitus/)

Medication adherence:

(Medication adherence.mp. OR medication compliance.mp OR medication persistence.mp.

OR medication concordance.mp. OR treatment adherence.mp. OR adherence.mp.)

Glycaemic control:

(Glycaemic control.mp. OR Glycated Haemoglobin A.mp. OR Hb A1c.mp. OR haemoglobin A1c.mp)

Ethnicity:

(Ethnic minority.mp. or ethnic group/ OR ethnicity.mp. OR White.mp. or Caucasian/ OR Asian American/ or Asian/ or British Asian/ or Asian.mp. or Asian continental ancestry group/ or South Asian/OR Black.mp. or Black person/ OR African American.mp. OR latino.mp. or Hispanic/OR exp Minority Groups/OR mixed race.mp. OR race.mp. OR exp "Latinos/Latinas"/ OR Mexican American.mp. OR European Continental Ancestry Group/ OR African Continental Ancestry Group/Arabs.mp. OR Maori.mp. or "Maori (people)" OR pacific.mp. OR Mexican American/ or Mexican/)

Qualitative methodology:

(Qualitative research/ or qualitative analysis/ or qualitative.mp. OR Focus group.mp. OR interviews.mp. OR grounded theory.mp. OR Action research.mp. OR content analysis.mp. OR thematic analysis.mp. OR NVivo.mp. OR Nudist.mp. OR Ethnology.mp.OR Perceptions.mp. OR Attitudes.mp. OR Views.mp. OR Ethnographic.mp. OR Themes.mp. OR Encounters.mp. OR Experiences.mp. OR meta ethnography.mp. OR qualitative study.mp. OR exp Qualitative Methods/)

Barriers or facilitators:

(barriers.mp. OR obstacles.mp. OR facilitators.mp.)

1. **Global health database**

Full search = (Diabetes) AND (Medication adherence) AND (Qualitative methodology) OR (Ethnicity)

Diabetes:

(Diabetes OR diabetes mellitus OR type 1 diabetes OR insulin dependent diabetes mellitus OR type 2 diabetes OR non-insulin dependent diabetes mellitus)

Medication adherence:

(Medication adherence OR medication compliance OR medication persistence

OR medication concordance OR treatment adherence OR adherence)

Qualitative methodology:

(Qualitative research OR qualitative analysis OR qualitative OR qualitative study OR Focus group OR interviews)

Ethnicity:

(Ethnic minority OR ethnic group OR ethnicity OR Minority Groups OR Asian American OR Asian OR South Asian OR Black OR African American OR Latino OR Hispanic OR Mexican American OR Arab)

1. **Grey literature search terms**

(Diabetes And Qualitative Study And Medication adherence And/or Ethnicity)
