# Supplementary material for: Understanding the influence of ethnicity on adherence to antidiabetic medication: Meta-ethnography and systematic review
Source: PLoS One. 2023 Oct 12;18(10):e0292581. doi: 10.1371/journal.pone.0292581 (PMC10569585; doi:10.1371/journal.pone.0292581)
Supplement: Attachment — Submitted filename: Peer review comments.docx [file pone.0292581.s003.docx]

**Introduction**

1. ‘’Across a number of high-income countries, lower rates of adherence to antidiabetic medications has been reported amongst minority ethnic groups. [12-16]’’; Such as…….

**Methods**

**Database**:

1. I think the list of databases is not complete. Other relevant database such as Global Health was not searched – that could potentially yield relevant articles for this review. Is there any specific reason why this database was not searched?

**Study selection and screening:**

**1.** What platform (s) did the authors use for screening of articles? This should be stated.

2. For rigour and transparency two independent reviewers are expected to screen titles and abstracts. Why did only one reviewer screen titles and abstracts?

3. ‘’The screenings of the articles’ full text were undertaken by RA and checked in full by authors AKH and AR-B.’’ This statement is not clear. Did the named authors independently screen the full text articles? If so, this should be explicitly stated.

**Study reading, data extraction and quality appraisal**

1. Two reviewers should independently assess the quality of systematic review using the Assessing the Methodological Quality of Systematic Reviews (AMSTAR) tool – this should be submitted with the manuscript.
2. Did authors work independently to extract data, or was any process in place for obtaining or confirming data from study investigators?
3. Confidence in the evidence: The authors should use the Confidence in the Evidence from Reviews of Qualitative research (CERQual) tool to assess the confidence of the key findings of this review. The authors could report or summarise findings in a table as part of review.

Discussion

1. Strengths and limitations of the review should have a separate subheading.
2. The authors should provide a separate subheading under the discussion section to discuss in detail the implications of their findings for practice, public health and future research.
